# Transcriptomic Profiling of Tetrodotoxin-Induced Neurotoxicity in Human Cerebral Organoids

**DOI:** 10.3390/md21110588

**Published:** 2023-11-10

**Authors:** Zhanbiao Liu, Zhe Wang, Yue Wei, Jingjing Shi, Tong Shi, Xuejun Chen, Liqin Li

**Affiliations:** 1 State Key Laboratory of NBC Protection for Civilian, Beijing 102205, Chinashijingjing9287@126.com (J.S.);; 2 Key Laboratory of Biotechnology and Bioresources Utilization of Ministry of Education, Dalian Minzu University, Dalian 116600, China

**Keywords:** tetrodotoxin, human cerebral organoids, transcriptomic profiling

## Abstract

Tetrodotoxin (TTX) is an exceedingly toxic non-protein biotoxin that demonstrates remarkable selectivity and affinity for sodium channels on the excitation membrane of nerves. This property allows TTX to effectively obstruct nerve conduction, resulting in nerve paralysis and fatality. Although the mechanistic aspects of its toxicity are well understood, there is a dearth of literature addressing alterations in the neural microenvironment subsequent to TTX poisoning. In this research endeavor, we harnessed human pluripotent induced stem cells to generate cerebral organoids—an innovative model closely mirroring the structural and functional intricacies of the human brain. This model was employed to scrutinize the comprehensive transcriptomic shifts induced by TTX exposure, thereby delving into the neurotoxic properties of TTX and its potential underlying mechanisms. Our findings revealed 455 differentially expressed mRNAs (DEmRNAs), 212 differentially expressed lncRNAs (DElncRNAs), and 18 differentially expressed miRNAs (DEmiRNAs) in the TTX-exposed group when juxtaposed with the control cohort. Through meticulous Gene Ontology (GO) annotation, Kyoto Encyclopedia of Genes and Genomes (KEGG) enrichment analysis, and protein–protein interaction (PPI) analysis, we ascertained that these differential genes predominantly participate in the regulation of voltage-gated channels and synaptic homeostasis. A comprehensive ceRNA network analysis unveiled that DEmRNAs exert control over the expression of ion channels and neurocytokines, suggesting their potential role in mediating apoptosis.

## 1. Introduction

Tetrodotoxin (TTX) stands as a highly potent nonprotein natural toxin, ubiquitously present across diverse aquatic and terrestrial species [1]. Originally identified in pufferfish in 1964, TTX has since been detected in a wide array of aquatic and terrestrial organisms. This neurotoxin operates as a swift and reversible inhibitor of sodium channels, exerting its action primarily on the central nervous system. Specifically, TTX selectively blocks sodium channels, impeding the ingress of sodium ions into cells and perturbing the initiation of cellular membrane action potentials. Consequently, this inhibition culminates in the dampening of excitatory processes in nerves and muscles. In human beings, TTX exposure can manifest as limb paralysis, paresthesia, and even cardiac failure [2,3], often leading to fatality within a matter of hours in cases of acute intoxication. Unfortunately, due to the absence of effective antidotes, treatment options are confined to symptom-based supportive care [4]. Conversely, owing to its exceptional selectivity and potent influence on voltage-gated sodium channels (VGSC), TTX has emerged as a pivotal tool in VGSC research. This unique attribute bestows TTX with significant potential in diverse applications, encompassing local anesthesia, analgesia, arrhythmia therapy, and addiction mitigation [5,6,7,8].

The toxicological mechanisms of tetrodotoxin (TTX) have primarily been elucidated through classical animal models. However, concerns have been raised regarding the translatability of findings from animal-based studies due to inherent physiological, genetic, and developmental disparities between human and animal brains. In recent years, as biomedical technology has progressed, cerebral organoids have emerged as promising in vitro models for replicating the complexities of the human nervous system and for toxicity screening [9]. Cerebral organoids represent three-dimensional organ-like cultures derived from human induced pluripotent stem cells (iPSCs) or embryonic stem cells (ESCs). Unlike conventional two-dimensional cell cultures, which often lack cellular diversity due to a uniform cell-type composition, cerebral organoids faithfully replicate the molecular, cellular, structural, and functional attributes of the human brain [10]. Consequently, cerebral organoid technology not only holds substantial promise for faithfully recapitulating human neurodevelopment and disease but also serves as a powerful tool for investigating toxicant-induced toxicity and underlying mechanisms [11,12].

For instance, iPSC-derived cerebral organoids have been employed to emulate the impact of serum exposure on blood–brain barrier disruption in Alzheimer’s disease patients [13]. Furthermore, cerebral organoids generated from iPSCs derived from microcephaly patients have consistently exhibited significantly reduced sizes, thus serving as an early testament to the capacity of cerebral organoids to manifest neurodevelopmental disorder phenotypes [14]. These versatile models are also instrumental in elucidating viral tropism, infection dynamics, their effects on organoid function, size, and cellular composition, as well as innate immune responses. Notably, cerebral organoids have made substantial contributions to unraveling the pathophysiology of neurotropic viral infections and evaluating the efficacy of antiviral agents within physiologically relevant models [15]. In consonance with these strides, our research endeavors focus on employing iPSC-derived cerebral organoids as a model to probe hitherto unknown toxicological mechanisms ensuing from exposure to tetrodotoxin.

Whole transcriptome sequencing has emerged as a widely employed tool in the fields of pharmacology and toxicology, facilitating comprehensive investigations into molecular pathways. This technique encompasses the sequencing of multiple RNA species, encompassing messenger RNA (mRNA), microRNA (miRNA), circular RNA (circRNA), and long non-coding RNA (lncRNA). mRNAs harbor specific genetic information, while miRNAs can interact with target mRNAs to degrade them or inhibit their translation. Despite the absence of protein-coding capacity, lncRNAs wield significant influence over gene expression through cis and trans-regulatory mechanisms. The ceRNA network, a novel gene regulation system comprising interactions between lncRNA, miRNA, and mRNA, has been implicated in the pathogenesis of numerous disorders. The application of transcriptomics in toxicological investigations has gained prominence. Our whole-transcriptome analysis revealed that the downregulation of specific genes precipitates apoptosis following a 24 h exposure to 10 μM TTX.

Notably, there is a dearth of existing literature concerning whole-transcriptome studies conducted in organoid models or pertaining to the ceRNA regulatory network implicated in TTX-induced brain injury and alterations in synaptic homeostasis.

## 2. Results

### 2.1. Cerebral Organoid Formation and Cultivation

The generation of cerebral organoids from iPSCs involves four distinct stages: embryoid body (EB) formation, induction, neuroepithelial expansion, and maturation. On the initial day, human iPSCs autonomously aggregated into EBs, attaining diameters of approximately 100 to 200 μm, which subsequently expanded to approximately 300 to 400 μm by the conclusion of the EB formation stage. Upon the transition to induction medium on day 5, the cultured tissues exhibited diameters of nearly 500 μm. These developed tissues were encapsulated within Matrigel droplets and transferred to a 6-well ultra-low-adherence cell culture plate for continued expansion in expansion media. On day 10, the cultured tissues were transitioned to a maturation medium and placed on a low-speed orbital shaker. By approximately day 40, these cultured tissues matured into cerebral organoids with diameters of approximately 3 to 5 mm, concurrently revealing the presence of dispersed cortical cells.

**Figure 1:**
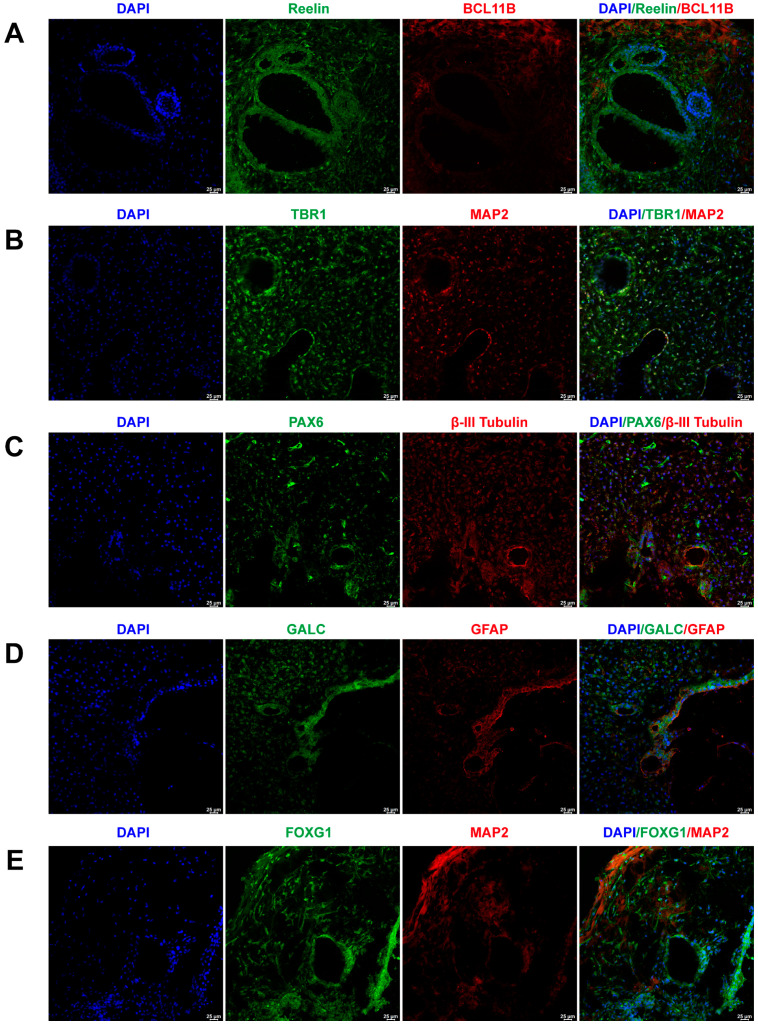
Generation and characterization of human-induced pluripotent stem cell (iPSC)-derived brain organoids. (**A**) Immunofluorescent staining of cerebral organoids using BCL11B and Reelin antibodies, demonstrating the presence of early-born layer 5 and the superficial cortex, respectively. (**B**) Immunofluorescent staining of cerebral organoids using MAP2 and TBR1 antibodies, confirming the expression of neurons and layer 6 neurons, respectively. (**C**) Immunofluorescent staining of cerebral organoids using PAX6 and βIII-tubulin antibodies, indicating the expression of radial glial cells and neurons, respectively. (**D**) Immunofluorescent staining of cerebral organoids using GFAP and GALC antibodies, revealing the expression of astrocytes and oligodendrocytes, respectively. (**E**) Immunofluorescent staining of cerebral organoids using FOXG1 and MAP2 antibodies, confirming the expression of forebrain and neurons, respectively. Scale bar = 25 μm.

To assess the representation of major brain cell types within the cerebral organoids, their markers were identified through immunofluorescence staining. Figure 1A illustrates the positive expression of early-born layer 5 and superficial cortex markers, BCL11B and Reelin, in cerebral organoids. Figure 1B depicts the positive expression of neurons and layer 6 neuron markers, TBR1 and MAP2, in cerebral organoids. Figure 1C exhibits the co-expression of PAX6 and βIII-Tubulin, markers for radial glial cells and neurons, within cerebral organoids. The co-expression of astrocyte and oligodendrocyte markers, GFAP and GALC, in cerebral organoids is presented in Figure 1D. Furthermore, cerebral organoids also featured neurons positive for forebrain markers FOXG1 and MAP2, as shown in Figure 1E.

### 2.2. Tetrodotoxin Exposure Causes Changes in Cerebral Organoid Cell Viability

**Figure 2:**
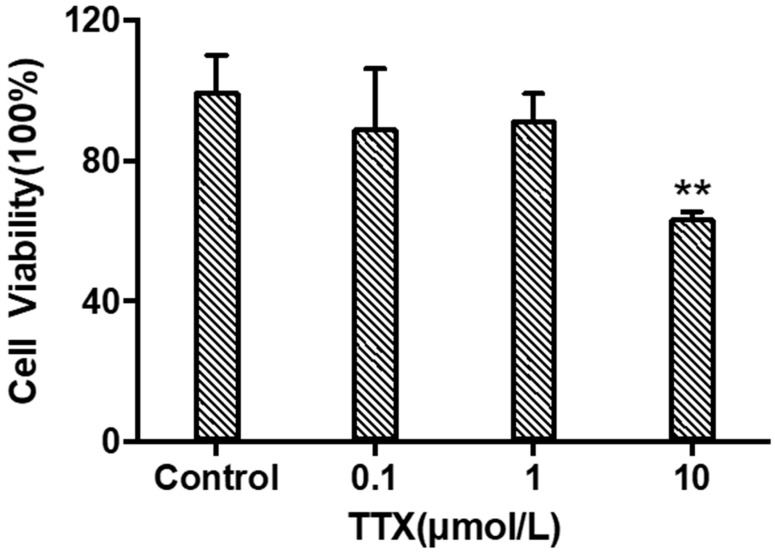
Viability of cerebral organoid cells under different concentrations of TTX (mean ± SD, *n* = 3; ** *p* < 0.01).

When TTX was incubated with cerebral organoids at concentrations of 0.1 μM, 1 μM, and 10 μM for 24 h. As shown in Figure 2, the cell viability of each group was (89.39 ± 16.80)%, (91.73 ± 7.38)%, and (63.72 ± 1.73)% of the normal control group, respectively. TTX had significant toxicity to cerebral organoids at a concentration of 10 μM (*p* < 0.05). Therefore, a concentration of 10 μM was selected for omics analysis and considering the construction of a neurotoxicity model.

### 2.3. Tetrodotoxin Exposure Induces Disruption in RNA Expression within Cerebral Organoids

**Figure 3:**
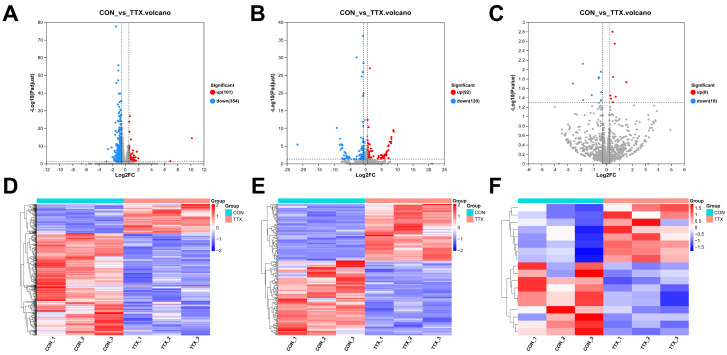
Analysis of differentially expressed RNAs (DERNAs) and heatmap comparison in TTX-treated and control samples. (**A**) Volcano plot illustrating differentially expressed mRNAs (DEmRNAs), where blue dots represent downregulated RNAs, red dots signify upregulated RNAs, and gray dots indicate RNAs with no significant differences compared to the control group. (**B**) Volcano plot of differentially expressed long noncoding RNAs (DElncRNAs). (**C**) Volcano plot of differentially expressed miRNAs (DEmiRNAs). (**D**) Hierarchical clustering and heatmap analysis of DEmRNAs, with red denoting upregulated RNAs and blue representing downregulated RNAs compared to the control group. (**E**) Hierarchical clustering and heatmap analysis of DElncRNAs. (**F**) Hierarchical clustering and heatmap analysis of DEmiRNAs, where red indicates upregulated RNAs and blue indicates downregulated RNAs compared to the control group.

To elucidate the molecular mechanisms underlying TTX-induced cytotoxicity in cerebral organoids, comprehensive transcriptional sequencing was employed to analyze RNA expression within the brain. mRNA and lncRNA were subjected to stringent screening criteria (|log2FC|≥1.5, *p*-adjust < 0.05). This analysis unveiled 455 differentially expressed mRNAs (DEmRNAs), comprising 101 upregulated and 354 downregulated transcripts (Figure 3A). Additionally, 212 differentially expressed lncRNAs (DElncRNAs) were identified, encompassing 92 upregulated and 120 downregulated transcripts (Figure 3B). miRNA analysis applied the following criteria (|log2FC|≥ 1.2, *p* < 0.05), identifying a total of 18 differentially expressed miRNAs (DEmiRNAs), featuring 8 upregulated and 10 downregulated miRNAs (Figure 3C) (Supplementary Table S1). Notably, heat map analysis distinctly demonstrated clustering and segregation between the control group and tetrodotoxin-exposed group, affirming the reliability of differential expression analysis (Figure 3D–F). These findings underscore the significant impact of tetrodotoxin exposure on RNA expression within cerebral organoids.

### 2.4. Tetrodotoxin Exposure Results in Dysregulated RNA Enrichment in GO and KEGG Pathways

**Figure 4:**
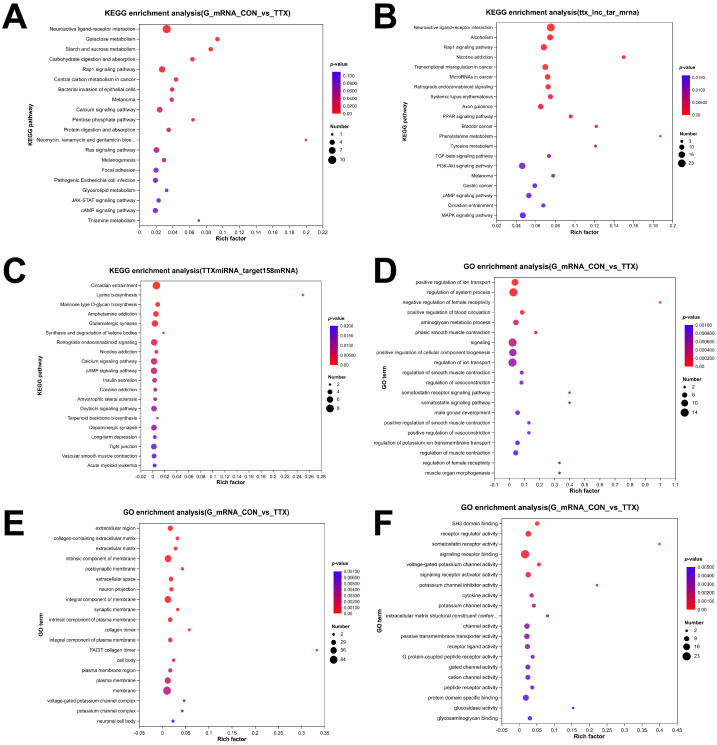
Functional enrichment analysis of dysregulated RNAs. (**A**–**C**) Top 20 dysregulated RNAs KEGG pathway enrichment analysis: (**A**) KEGG pathway enrichment analysis of differentially expressed mRNAs (DEmRNAs). (**B**) KEGG pathway enrichment analysis of mRNAs targeted by differentially expressed long noncoding RNAs (DElncRNAs). (**C**) KEGG pathway enrichment analysis of mRNAs targeted by differentially expressed miRNAs (DEmiRNAs). (**D**–**F**) Top 20 Gene Ontology (GO) enrichment analysis: (**D**) GO enrichment analysis of biological processes. (**E**) GO enrichment analysis of cellular components. (**F**) GO enrichment analysis of molecular function. The dot size corresponds to the number of enriched genes, and the color gradient indicates the level of enrichment significance, ranging from blue to red.

To gain insight into the functional repercussions of the aforementioned dysregulated RNAs, we conducted GO enrichment and KEGG pathway analyses, employing a significance threshold of *p* < 0.05. The analysis yielded a total of 732 significantly enriched GO terms within DEmRNAs, encompassing 530 biological process (BP) terms, 128 molecular function (MF) terms, and 74 cellular component (CC) terms. In BP analysis, DEmRNAs were prominently associated with ion transport, blood circulation, phasic smooth muscle contraction, and cellular component biogenesis (Figure 4D). CC analysis indicated high enrichment of DEmRNAs in the extracellular region, extracellular matrix, postsynaptic membrane, neuron projection, and synaptic membrane (Figure 4E). MF analysis revealed significant enrichment of DEmRNAs in functions such as SH3 domain binding, receptor regulator activity, somatostatin receptor activity, signaling receptor binding, and voltage-gated potassium channel activity (Figure 4F).

To validate the involved signaling cascades, KEGG pathway analysis was conducted, and the top 20 highly enriched pathways were visualized in a bubble plot. These pathways included neuroactive ligand–receptor interaction, calcium signaling pathway, JAK-STAT signaling pathway, and cAMP signaling pathway (Figure 4A), among others. Furthermore, KEGG analysis of DElncRNAs (Figure 4B) and DEmiRNAs (Figure 4C), expected to target genes, indicated that the enriched pathways primarily revolved around neuroactive ligand–receptor interaction, calcium signaling pathway, glutamatergic synapse, dopaminergic synapse, vascular smooth muscle contraction, and other relevant processes.

### 2.5. Functional Analysis of the Protein–Protein Interaction (PPI) Network

**Figure 5:**
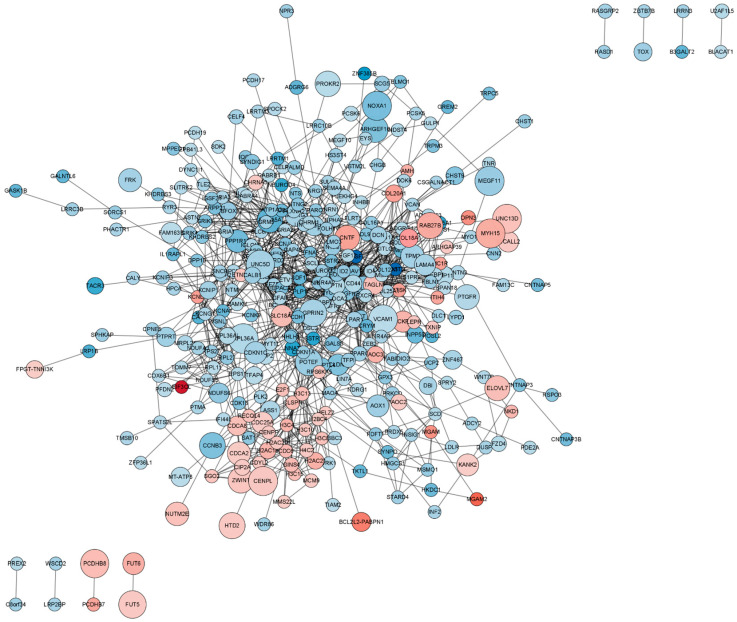
Protein–protein interaction (PPI) network of differentially expressed mRNAs (DEmRNAs). In this network visualization, upregulated DEmRNAs are depicted as red nodes, while downregulated DEmRNAs are represented as blue nodes. The connecting lines between nodes illustrate the interactions among proteins.

**Figure 6:**
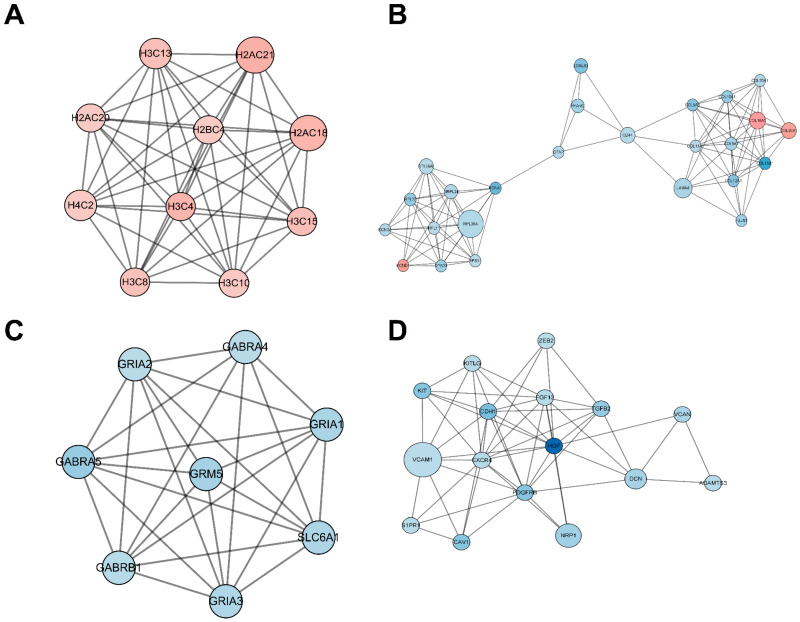
Significantly clustered modules from the protein–protein interaction (PPI) network. Within this figure, we depict four distinct modules derived from the PPI network analysis. Upregulated differentially expressed mRNAs (DEmRNAs) are visualized as red nodes, while downregulated DEmRNAs are represented as blue nodes. The connections between nodes signify protein–protein interactions.

The PPI network encompassing all DEmRNAs from both the control and TTX exposure groups was constructed using the String database to elucidate the interconnections among these DEmRNAs, as illustrated in Figure 5. Cytoscape was employed for PPI network inspection, while the MCODE plug-in of Cytoscape was utilized to generate sub-networks, revealing hub modules and pivotal genes within the PPI network. We emphasize the four modules characterized by the most extensive interactions in the ensuing subsection. Module 1 (score = 10, Figure 6A) consists of 10 nodes and 45 interaction pairings, with the associated genes primarily engaged in systemic lupus erythematosus, alcoholism, and necroptosis. Module 2 (score = 8.25, Figure 6B) comprises 25 nodes and 99 interaction pairings, with the connected genes predominantly enriched in protein digestion and absorption, ribosome ECM–receptor interaction, and neuroactive ligand–receptor interaction. Module 3 (score = 7.714, Figure 6C) encompasses 8 nodes and 27 interaction pairings, with the genes chiefly linked to nicotine addiction, GABAergic synapse, morphine addiction, retrograde endocannabinoid signaling, and taste transduction. Module 4 (score = 7.2, Figure 6D) includes 16 nodes and 54 interaction pairings, with the genes concentrated predominantly in cell adhesion molecules, Rap1, Ras, and MAPK signaling pathways. The pathways involved in these core nodes are primarily associated with synaptic homeostasis and synaptic plasticity. As TTX affects the cell membrane’s action potential, it subsequently influences the postsynaptic membrane’s response to neurotransmitters.

### 2.6. Competitive Endogenous RNA (ceRNA) Network of DEmRNAs, DElncRNAs, and DEmiRNAs

The subcellular localization of DElncRNAs was initially predicted using the http://www.csbio.sjtu.edu.cn/bioinf/lncLocator/ (accessed on 5 September 2023) website, resulting in the identification of 14 cytoplasmic lncRNAs (Supplementary Table S1). These lncRNAs can serve as microRNA sponges or miRNA precursors, thereby modulating miRNA expression and function. Subsequently, the Targetscan and Miranda algorithms were employed to determine target mRNAs and lncRNAs of DEmiRNAs. The acquired target mRNAs and lncRNAs were then combined with DEmRNAs and DElncRNAs, respectively. This comprehensive analysis yielded 24 DEmRNAs and 15 lncRNAs, along with 27 miRNA–mRNA interaction pairs and 28 lncRNA–miRNA interaction pairs. Consequently, a biologically relevant lncRNA–miRNA–mRNA ceRNA network was constructed.

**Figure 7:**
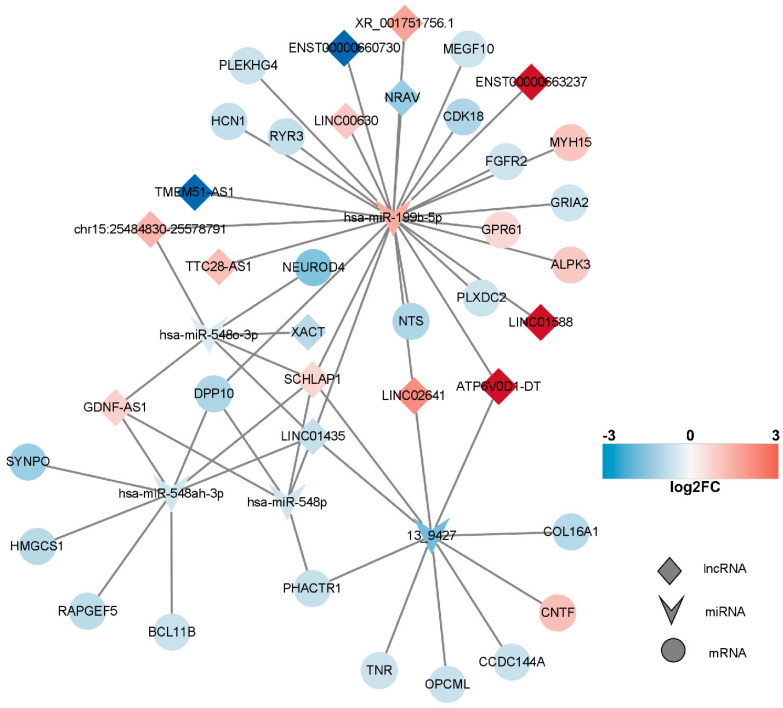
lncRNA–miRNA–mRNA network. In this figure, the network comprising long non-coding RNAs (lncRNAs), microRNAs (miRNAs), and messenger RNAs (mRNAs) is presented. Upregulated RNAs are depicted in red, while downregulated RNAs are represented in blue. Circular nodes symbolize mRNAs, prism nodes denote miRNAs, and triangular nodes signify lncRNAs.

**Figure 8:**
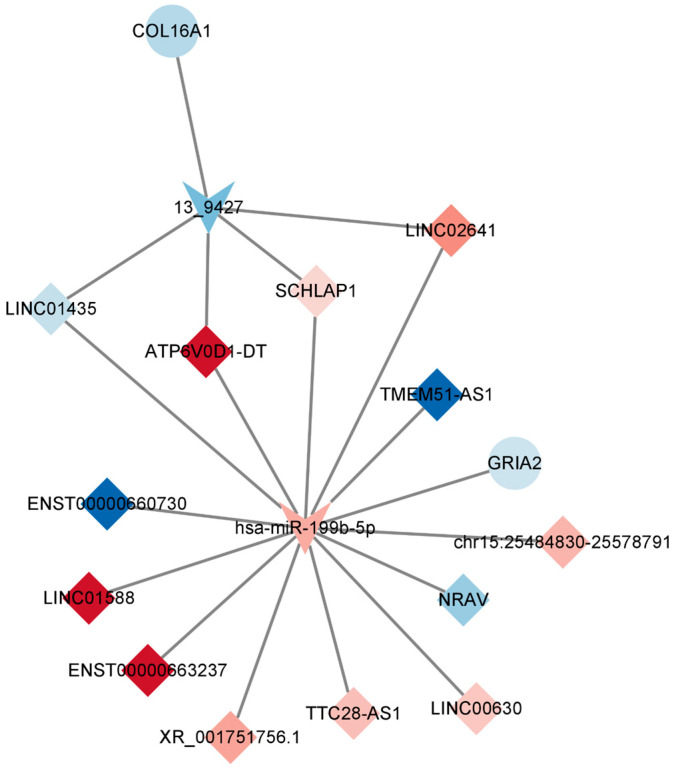
Key lncRNA–miRNA–mRNA ceRNA subnetwork. This figure illustrates the key competing endogenous RNA (ceRNA) subnetwork involving long non-coding RNAs (lncRNAs), microRNAs (miRNAs), and messenger RNAs (mRNAs). In this representation, prism nodes signify lncRNAs, arrow nodes symbolize miRNAs, and circular nodes represent mRNAs.

In line with the ceRNA hypothesis, common miRNAs acted as binding sites, with upregulated miRNAs associated with downregulated lncRNAs and mRNAs, and downregulated miRNAs linked to upregulated lncRNAs and mRNAs. The down–up–down network comprised 3 lncRNAs, 1 miRNA, and 11 mRNAs, resulting in 15 nodes and 26 edges (Figure 7, left panel). Additionally, the up–down–up network, comprising 1 node and 10 edges (Figure 8), consisted of three lncRNAs, one miRNA, and one mRNA.

### 2.7. Construction of the Key lncRNA–miRNA–mRNA Subnetwork

In order to construct a pivotal ceRNA subnetwork that effectively captures the functions of the aforementioned ceRNA network, we established a network comprising 13 lncRNAs, 2 miRNAs, and 2 mRNAs, resulting in 17 nodes and 19 edges. Notably, the downregulated gene GRIA2 encodes the glutamate ion receptor AMPA-type subunit 2. It can be inferred that TTX exerts its impact by impeding sodium ions from entering cells, consequently affecting the generation of cell membrane action potentials. Neurons rely on the release of neurotransmitters for signal conduction and output, and this release is facilitated by numerous vesicles during cell depolarization stimulation. Applying electrical stimuli to nerve cells induces the release of neurotransmitters, resulting in eEPSC or eIPSC. As the occupation of sodium channels by tetrodotoxin is reversible, synaptic homeostasis is restored when sodium channels regain their functionality.

### 2.8. TTX-Induced Alterations in Postsynaptic Membrane Homeostasis

Previous studies have indicated that TTX inhibits synaptic strengthening that depends on PSD-95 [16,17,18]. Exposure to TTX disrupts the equilibrium of neuroligand interactions, modifies postsynaptic membrane homeostasis, and leads to a decrease in PSD-95 expression.

**Figure 9:**
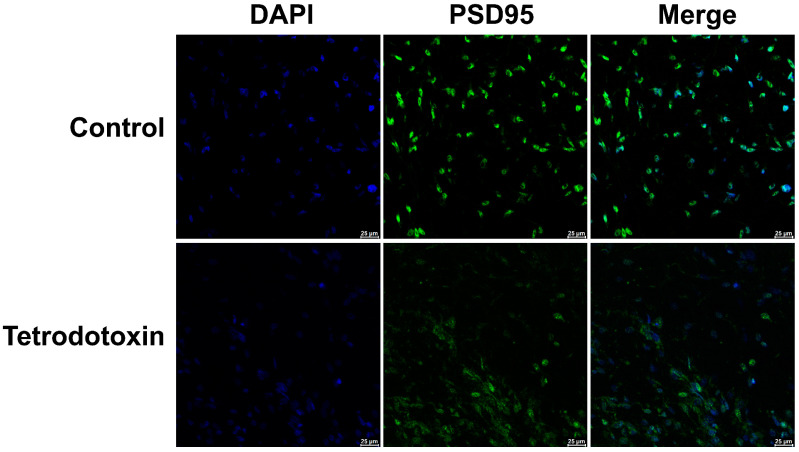
Fluorescent image depicting PSD-95 staining in organoids. The fluorescent micrograph displaying the staining of PSD-95 (postsynaptic density protein 95, a marker for the postsynaptic membrane) in organoids. PSD-95 is represented in green, while cell nuclei are shown in blue. The images vividly illustrate the reduction in PSD-95 expression on the postsynaptic membrane of cerebral organoids following exposure to tetrodotoxin. Scale bar = 25 μm.

Based on the existing omics data, we postulate that tetrodotoxin influences the synaptic homeostasis of cerebral organoids and impacts glutaminergic synapses, resulting in alterations in synaptic homeostasis. Immunofluorescent staining of cerebral organoid sections has demonstrated a reduction in the expression of PSD-95 protein. After 24 h of incubation with tetrodotoxin, PSD-95 expression was found to be decreased in cerebral organoids (Figure 9). This observation aligns with prior research findings.

**Figure 10:**
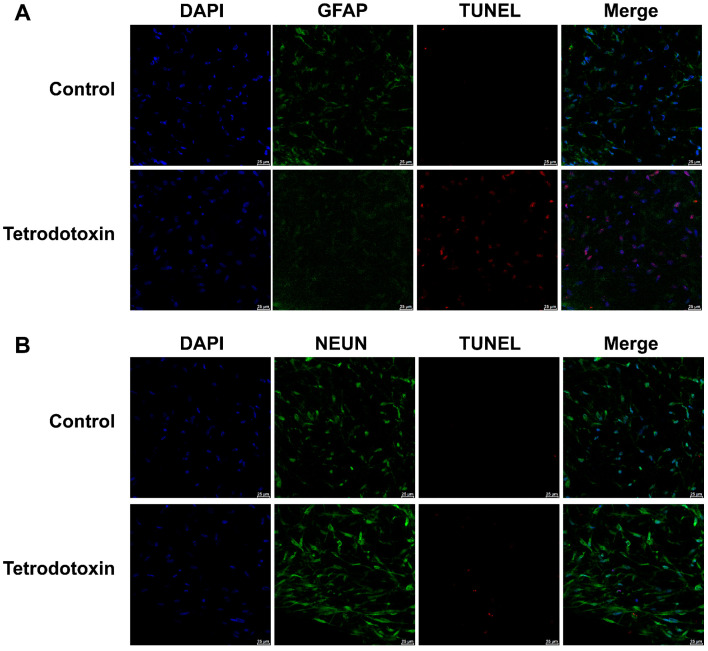
Fluorescent images of GFAP and TUNEL co-staining in organoids. (**A**) Cerebral organoids were either untreated or treated with 10 nM tetrodotoxin for 24 h. Nuclei were stained with DAPI and are represented in blue, while GFAP and TUNEL signals are depicted in green and red, respectively. (**B**) Fluorescent micrographs of cerebral organoids subjected to NeuN and TUNEL co-staining. The staining highlights NeuN (green), TUNEL signal (red), and cell nuclei (blue). The scale bar measures 25 μm.

Furthermore, we conducted immunofluorescence staining for astroglial cell markers, neuronal cell markers, and TUNEL assays in both the exposed and control groups. As shown in Figure 10A,B, following exposure to tetrodotoxin, astrocytes and neurons exhibited partial apoptosis. This apoptosis may arise due to disruptions in synaptic homeostasis.

## 3. Discussion

TTX exerts its toxic effects by selectively targeting voltage-gated sodium channels (Nav) [19,20]. Clinical manifestations of TTX intoxication encompass symptoms such as tingling of the tongue and lips, perioral abnormalities, quadriplegia or numbness, and muscular incoordination. In severe cases, poisoning can progress to respiratory paralysis and cardiac arrest, potentially leading to shock or fatality due to insufficient oxygen delivery to the brain. Our finding represents a pioneering effort that harnesses iPSC-derived cerebral organoids, bridging the gap between TTX exposure analysis in animal models and its implications in human systems. Our findings illuminate the multi-tiered neurotoxic effects induced by TTX in human cerebral organoids, encompassing alterations in tissue structure, cellular activity, gene expression patterns, and regulatory networks. Immunofluorescence analyses revealed the occurrence of apoptosis in cerebral organoids within 24 h of TTX exposure.

Gene Ontology (GO) annotations unveiled the involvement of DEmRNAs in processes such as positive regulation of ion transport, synaptic membrane organization, regulation of blood circulation, and neuron projection development. These DEmRNAs significantly impact receptor modulatory activity, signaling receptor binding, voltage-gated potassium channel activity, and potassium channel inhibitor activity. These findings underscore the influence of TTX exposure on the expression levels of genes central to synaptic function and homeostasis. KEGG enrichment analysis revealed that the primary targets of DEmRNAs, DElncRNAs, and DEmiRNAs included pathways such as neuroactive ligand–receptor interaction, Rap1 signaling pathway, calcium signaling pathway, glutamatergic synapse, long-term depression, and the cAMP signaling pathway. Glutamate synapses serve as the principal excitatory synapses in the human brain and constitute the structural foundation for synaptic plasticity and the regulation of synaptic homeostasis [21]. The enriched pathways chiefly revolve around neurotoxicity, neurotransmitter conduction, voltage gating, and analgesic withdrawal.

Subsequently, we assembled a protein–protein interaction (PPI) network incorporating all DEmRNAs, culminating in a network featuring 346 nodes and 1189 edges. Following this, we employed the MCODE plug-in within the Cytoscape software (v3.9.1) to identify four modules characterized by the closest interactions. These major nodes predominantly intersect with pathways crucial for synaptic homeostasis and synaptic plasticity.

When tetrodotoxin contacts cerebral organoids, the top four nodes with the highest scores can be seen through the PPI network, as shown in Figure 5C. GABRA5 and Grm5 in this subnetwork are gamma-aminobutyric acid type A receptor subunit alpha5 and glutamate metabotropic receptor 5. Downregulation of GABRA5 can have multiple effects on metabolism and physiology. GABRA5 is a subunit of the GABAA receptor involved in inhibitory neurotransmission in the brain. Downregulation of GABRA5 may result in impaired inhibitory neurotransmission and reduced number or function of GABAA receptors, resulting in decreased inhibitory neurotransmission. This disrupts the balance between excitatory and inhibitory signals in the brain, potentially leading to increased neuronal excitability and altered neural network activity. GABRA5 downregulation may also have metabolic effects, but the specific mechanism is unclear. GABA receptors are involved in the regulation of glucose metabolism, insulin secretion, and energy homeostasis. Changes in GABAA receptor function may disrupt these metabolic processes [22]. Grm5 plays an important role in regulating synaptic plasticity, brain neurotransmitter levels, calcium signaling pathways in astrocytes, the ability to regulate neuroinflammation, and metabolic processes such as glucose uptake and lactate production in the brain. Downregulation of Grm5 may disrupt the normal function of synapses and impair synaptic plasticity [23,24]; affect the release of neurotransmitters, leading to an imbalance in excitatory and inhibitory signaling; disrupt calcium homeostasis and signaling, thus having widespread effects on cellular processes [25]; affect the ability of astrocytes to regulate neuroinflammation; and affect energy metabolism in the brain [26]. TTX-induced alterations in cell membrane action potential further impinge upon the responses of postsynaptic membranes to neurotransmitters.

Predictive analysis of target genes yielded 108 miRNA–mRNA interaction pairs, 33 miRNA–DElncRNA interaction pairs, and 6285 lncRNA–mRNA interaction pairs. On this foundation, we constructed a physiologically relevant ceRNA network. Upon exposure to tetrodotoxin, a noteworthy escalation in cellular apoptosis was observed. The underlying factors governing cell apoptosis are multifaceted, entailing the participation and regulation of various molecular components. In essence, these mechanisms can be categorized into two fundamental pathways: the extrinsic (death receptor) pathway and the intrinsic (mitochondria) pathway. Despite the substantial differences in the mechanisms and molecules involved in these two pathways, they are inherently interconnected [27,28].

The Ryanodine receptor (RYR), primarily associated with Ca^2+^ release from the endoplasmic reticulum (ER), has garnered less attention in its role in apoptosis regulation. RYR type II, in particular, has been implicated in mediating Ca^2+^ transfer from the sarcoplasmic reticulum (SR) to cardiac mitochondria via direct interaction with the mitochondrial voltage-dependent anion channel 2 (VDAC2) [29]. Inhibition of ER Ca^2+^ release through RYR has been demonstrated to safeguard cortical neurons against NMDA-induced excitotoxicity by curbing cytosolic Ca^2+^ surges and mitigating mitochondrial and ER stress [30]. The rapid onset of fatality resulting from TTX poisoning is evident, with systemic distribution evident in animal poisoning models. Our ceRNA network analysis unveiled the downregulation of the RYR3 gene, while its homologous genes within the RYR family predominantly govern intracellular Ca^2+^ release from calcium pools to the cytoplasm. Although the precise regulatory role of RYR3 in Ca^2+^ dynamics remains elusive, it is conceivable that it possesses the potential to elicit apoptosis akin to its family counterparts.

CNTF, conventionally recognized as a neuroprotective factor, is often associated with promoting cell survival and axonal regeneration. However, the downregulation of CNTF expression following exposure to TTX raises questions regarding its role in cell protection and apoptosis [31]. Conversely, the downregulation of BCL11B prompts the activation of PTK7, culminating in the inhibition of cell proliferation and the facilitation of apoptosis [32,33,34]. PHACTR1, while inhibiting endothelial cell proliferation and migration, augments apoptosis and oversees the regulation of matrix metalloproteinases and apoptosis-related proteins [35]. Inactivation or ectopic expression of OPCML precipitates cell cycle arrest and apoptosis, imparting profound effects on cell growth and proliferation across various malignancies, thus underscoring the potential of OPCML downregulation to induce apoptosis [36,37,38]. Neurotensin (NTS) serves as a neuroprotective agent against ischemic injury and harbors therapeutic potential. Within the central nervous system, NTS regulates neurotransmitter activities, particularly within the dopamine system. Its downregulation may disrupt neuropeptide transport, consequently impinging upon the normal release and functioning of neuropeptides and neurotransmitters [39]. CDK18, by activating the RAS/mitogen-activated protein kinase kinase 1/extracellular signal-regulated kinase pathway, fosters the differentiation of oligodendrocyte precursor cells without exerting influences on their proliferation or apoptosis [40].

The gene GRIA2, found to be downregulated in the ceRNA regulatory network following tetrodotoxin exposure, aligns with previously reported results [41]. This gene encodes the glutamate ion receptor AMPA-type subunit 2, sensitive to AMPA enzymes, and functions as a ligand for activating cation channels. Within the central nervous system, GRIA2 operates as an excitatory neurotransmitter receptor, playing a pivotal role in neuronal synaptic transmission. Its downregulation may impair normal neuronal function and hamper neurotransmitter conduction. Subsequently, the downregulation of the TNR gene, responsible for encoding an extracellular matrix glycoprotein exclusively expressed in the central nervous system, implies a role in regulating neurite outgrowth, neuronal adhesion, and sodium channel function. Furthermore, the HCN1 gene codes for the hyperpolarization-activated cyclic nucleotide-gated potassium channel 1, which also operates as a cation channel. Reduced expression of HCN1 may heighten neuronal excitability, given its inhibitory role in neurons. Diminished HCN1 channel expression can lead to heightened excitability, possibly facilitating the generation of action potentials. The decreased expression of HCN1 channels in presynaptic neurons may perturb synaptic transmission, resulting in heightened excitability or diminished postsynaptic neuron inhibition. HCN1 channels assume a pivotal role in the regulation of neuronal network activity. Therefore, a decrease in HCN1 channel expression might engender alterations in network activity patterns and frequencies. Notably, when exposed to 1 μM TTX for 48 h, an upregulation of HCN1 was observed, which may stem from the dynamic balance shifts initiated by the prior downregulation at 10 μM for 24 h. These intricate processes, involving the modulation of neurotransmitter conduction through promotion and inhibition of specific targets, highlight the synaptic homeostasis’s self-regulating potential. It follows that this self-regulation capability may hasten TTX’s inhibition of synaptic action potentials, offering a glimpse into the possibility of discovering an antidote [42].

Saxitoxin (STX), a sodium channel blocker similar to TTX, can also inhibit action potential firing and has been reported in many related transcriptome and proteome studies [43]. Chen et al. poisoned mouse neuroblastoma cells with low doses of STX and analyzed their proteomic changes. Subsequently, it was found that STX exposure increased the expression of six proteins and decreased the expression of three proteins. In addition, these nine proteins that were altered due to exposure to low-dose STX were mainly involved in apoptotic pathways, cytoskeleton maintenance, membrane potential, and mitochondrial function [44]. Sun et al. evaluated the neurotoxic effects of long-term low-dose STX exposure on C57/BL mice by analyzing the behavior of mice and the proteomic analysis of hippocampus after STX exposure. Multiple behavioral tests showed that mice at different doses developed cognitive deficits after 3 months. Compared with control mice, the neuronal cells in the CA1 area of the hippocampus were reduced and the pyramidal cell layer was thinned, resulting in brain neuron damage. A total of 29 proteins were significantly altered in different STX dose groups. Bioinformatics analysis showed that protein phosphatase 1 (Ppp1c) and arylsulfatase A (Arsa) are involved in the Hippo signaling pathway and the sphingolipid metabolism pathway. The decrease in Arsa expression indicates that long-term low-dose STX exposure can cause neuronal inhibition [45]. Our results on neurotoxicity induced by TTX on brain organoids are similar to these proteomic changes after STX exposure.

Taken together, these findings reveal novel mechanisms controlling neurotransmitter release and transmission at synapses, dynamic transmission balance, and apoptosis induced by TTX exposure in cerebral organoids models. However, the TTX concentration of 10 μM that we used to induce neurotoxicity in cerebral organoids is difficult to achieve in the human brain due to the existence of the blood–brain barrier in actual situations; these are still directions worthy of further research.

## 4. Materials and Methods

### 4.1. Materials

Human induced pluripotent stem cells (iPSCs) were procured from Beijing Cellapy Biotechnology Co., Ltd. (Beijing, China). The STEMdiff™ Brain Organoid Kit, mTeSRTM1 Complete Kit, and gentle cell dissociation reagent were sourced from STEMCELL Technologies (Vancouver, BC, Canada). The One Step TUNEL Apoptosis Assay Kit and DAPI were obtained from Shanghai Beyotime Biotechnology Co., Ltd. (Shanghai, China). CellTiter-Glo^®^ Luminescent Cell Viability Assay (CTG) was obtained from Promega (Beijing) Biotech Co., Ltd. (Beijing, China). Tetrodotoxin was provided by the Laboratory of Analytical Chemistry, Research Institute of Chemical Defence (Beijing, China). Monoclonal antibodies against BCL11B (1F8G8) and PAX6, as well as antibodies against Reelin, MAP2, β III Tubulin, GFAP, GALC, FOXG1, and PSD95, were procured from Invitrogen (Carlsbad, CA, USA) and Abcam (Cambridge, UK). Goat Anti-Mouse IgG H&L conjugated with AF594 was obtained from Beijing Bioss Biotechnology Co., Ltd. (Beijing, China), while Dylight 488 Goat Anti-Rabbit IgG(H+L) was sourced from Abbkine Scientific Co., Ltd. (Wuhan, China). Furthermore, 0.4% Trypan blue was acquired from Thermo Fisher Scientific Co. (Waltham, MA, USA).

### 4.2. Cultivation of 3D Cerebral Organoids Derived from iPSCs

The generation of 3D cerebral organoids from iPSCs was facilitated using the STEMdiff™ Brain Organoid Kit. In brief, iPSCs were dissociated with the gentle cell dissociation reagent in mTeSR^TM^1 at 37 °C for 12 min. Subsequently, cells were resuspended in embryoid body (EB) seeding media at a concentration of 8000 cells/mL and seeded into 96-well ultra-low adherent plates at a volume of 100 μL per well. EB formation media were added on days 2 and 4, followed by induction medium for a duration of 2 days. On day 7, the EBs were embedded in Matrigel droplets and cultured for an additional three days in a 6-well ultra-low adherent plate with expansion media. On day 10, the culture medium was replaced with maturation media, and the organoids were placed on an orbital shaker within a 37 °C incubator to support long-term cultivation. Cerebral organoids were sustained in culture for a period of up to 45 days.

### 4.3. Immunofluorescence Characterization of Cerebral Organoids

Following 45 days of cultivation, cerebral organoids were fixed in 4% paraformaldehyde at 4 °C for 24 h. Subsequently, the organoids were subjected to dehydration in a 30% sucrose aqueous solution at 4 °C for an additional 24 h. The gelatin-fixed cerebral organoids were sectioned into 15 μm thick sections, after which they underwent immunofluorescence labeling using a panel of primary antibodies, including BCL11B antibody, PAX6 monoclonal antibody, Anti-Reelin antibody, anti-MAP2 antibody, anti-βIII Tubulin antibody, anti-GFAP antibody, anti-GALC antibody, anti-FOXG1 antibody, and anti-PSD95 antibody. Secondary antibodies employed for fluorescence labeling included Goat Anti-Mouse IgG H&L/AF594 and Goat Anti-Rabbit IgG(H+L). Finally, the nuclei were stained with DAPI.

### 4.4. Treatment of Cerebral Organoids with Tetrodotoxin

#### 4.4.1. Cell Viability Assessment

The cell viability of cerebral organoids was detected using the CTG method. Cerebral organoids were incubated with TTX of 0 μM, 0.1 μM, 1 μM, and 10μM for 24 h, respectively. After the incubation, the medium was aspirated and washed three times with DPBS; then, 100 μL of medium was added and equilibrated at room temperature for 30 min. After equilibrium, 100 μL of CTG reagent was added, vortexed to completely lyse the cells, and incubated at room temperature for 30 min. Using a microplate reader, chemiluminescence detection was performed and the cell survival rate of cerebral organoids determined by quantifying ATP. The integration time was set to 1 s per well, the luminescence value of each well was read, and the cell viability rate of each group was calculated. Cell viability rate (%) = luminescence value of the TTX group/luminescence value of the normal control group × 100%.

#### 4.4.2. Cytotoxicity Assessment

The 3D cerebral organoids were exposed to either 0 μM (control) or 10 μM TTX for a duration of 24 h. Mature cerebral organoids were transferred into round-bottom 96-well plates for the drug administration group, wherein the existing culture medium was replaced with 100 μL of cerebral organoid culture medium containing 10 μM TTX per well. In the control group, 100 μL of cerebral organoid culture medium was similarly exchanged. Subsequently, both groups of cerebral organoids were cultured on a horizontal shaker within a sterile incubator, maintaining conditions at 37 °C and 5% CO_2_ for 24 h. Following incubation, the culture medium was discarded, and the organoids were gently rinsed five times with DPBS.

#### 4.4.3. TUNEL Assay

Cerebral organoid tissue slices derived from the control and TTX-exposed groups underwent investigation for cell apoptosis utilizing the One Step TUNEL Apoptosis Assay Kit. TUNEL labeling was conducted in conjunction with immunofluorescence staining to discern the various brain cell types experiencing apoptosis post TTX exposure. Primary antibodies targeting neuronal nuclear antigen (NeuN) or GFAP were employed, along with corresponding secondary antibodies (Alexa Fluor 488-conjugated mouse or rabbit IgG, Thermo Fisher Scientific). Cell nuclei were identified using DAPI, and imaging was carried out utilizing a Leica STELLARIS 5 confocal microscope.

### 4.5. Whole-Transcriptome Sequencing and Analysis

Total RNA extraction, library construction, and sequencing for both the tetrodotoxin-treated and control groups were conducted by Majorbio Bio-pharm Biotechnology Co., Ltd. (Shanghai, China). For detailed experimental protocols, please refer to Supplementary Materials. A whole-transcriptome sequencing and analysis was performed, which included three biological replicates.

To comprehensively analyze the differential expression of mRNAs, lncRNAs, and circRNAs across various samples and unravel the regulatory mechanisms of genes by integrating sequence functional information, RSEM (v3.3.2) software (http://deweylab.biostat.wisc.edu/rsem/, accessed on 24 June 2023) was employed for quantitative assessment of overall expression levels at the gene or transcript level for mRNA, lncRNA, and circRNA. By merging sequencing data with functional information, gene regulatory mechanisms were elucidated, and expression levels were standardized using the reads per million mapped reads (RPM) methodology. Subsequently, all differential expression analyses were conducted employing the DESeq2 program, with RNAs exhibiting |log2FC|>1.5 and *p*-value < 0.05 considered as significant differentially expressed RNAs (DERNAs).

The annotation of DERNAs and their target genes was accomplished through Gene Ontology (GO) and Kyoto Encyclopedia of Genes and Genomes (KEGG) database annotations. The top 20 most significantly enriched GO terms and KEGG pathways were employed to construct interaction networks for signaling pathways of DERNAs and their targets based on enrichment scores, at a significance level of *p* < 0.05 relative to the background encompassing all genes. GO annotations and KEGG pathway analyses were conducted utilizing Goatools (https://github.com/tanghaibao/Goatools, accessed on 16 July 2023) and KOBAS (http://kobas.cbi.pku.edu.cn/home.do, accessed on 16 July 2023). The protein–protein interaction (PPI) network was established based on DEmRNAs utilizing the STRING database (https://string-db.org/, accessed on 18 June 2023), and visualization and optimization were executed using Cytoscape software (https://cytoscape.org/, accessed on 26 June 2023). Module analysis of the PPI network was carried out using the MCODE plugin to identify meaningful interaction modules and cluster scores.

In accordance with the miRNA binding sites within the sequence of mRNA reaction components, TargetScan (http://www.targetscan.org/, accessed on 22 June 2023) and Miranda (http://www.miranda.org/, accessed on 22 June 2023) software were employed to analyze miRNA–mRNA and miRNA–lncRNA binding sites. The connections between lncRNA–miRNAs and miRNA–mRNAs were discerned, and a regulatory network encompassing circRNA–miRNA–mRNA interactions was established using miRNAs as pivotal components. DElncRNA, DEmiRNA, and DEmRNAs were scrutinized for their functional roles and mutual interactions. Finally, the competing endogenous RNA (ceRNA) network was visualized employing Cytoscape software (v3.9.1) [46].

## 5. Conclusions

This study introduces human cerebral organoids generated from iPSCs as a novel model for investigating the toxicological mechanisms of TTX. Exposure of cerebral organoids to TTX led to observed apoptosis. In-depth analysis of the regulatory relationships among differential RNAs culminated in the examination of the lncRNA–miRNA–mRNA network. These findings shed light on novel mechanisms governing neurotransmitter release and delivery at synapses, dynamic transmission equilibrium, and homeostasis restoration following TTX exposure within cerebral organoid models.

## Data Availability

The data presented in this study are available on request from the corresponding author.

## Supplementary Materials

The following supporting information can be downloaded at: https://www.mdpi.com/article/10.3390/md21110588/s1, Supplementary Methods: Characterizing Tetrodotoxin-Induced Neurotoxicity in Human Cerebral Organoids: Insights into Molecular Basis and Regulatory Networks; Supplementary Table S1: Details of mRNA, lncRNA and miRNA differential expression.
